# Training Indoor and Scene-Specific Semantic Segmentation Models to Assist Blind and Low Vision Users in Activities of Daily Living

**DOI:** 10.1109/OJEMB.2025.3607816

**Published:** 2025-09-09

**Authors:** Ruijie Sun, Giles Hamilton-Fletcher, Sahil Faizal, Chen Feng, Todd E. Hudson, John-Ross Rizzo, Kevin C. Chan

**Affiliations:** Computer Science Department, Courant Institute of Mathematical SciencesNew York University 5894 New York NY 10012 USA; Departments of Rehabilitation Medicine, Radiology, and Tech4Health Institute, NYU Grossman School of Medicine, NYU Langone HealthNew York University 5894 New York NY 10017 USA; Department of Computer Science and Engineering, Tandon School of EngineeringNew York University 5894 New York NY 10012 USA; Department of Civil and Urban Engineering & Department of Mechanical and Aerospace Engineering, Tandon School of EngineeringNew York University 5894 Brooklyn NY 11201 USA; Department of Rehabilitation Medicine, NYU Grossman School of Medicine, NYU Langone HealthNew York University 5894 New York NY 10017 USA; Departments of Rehabilitation Medicine, Neurology, and Ophthalmology, NYU Grossman School of Medicine, NYU Langone Health, & Department of Biomedical Engineering, Tandon School of EngineeringNew York University 5894 New York NY 10017 USA; Departments of Radiology and Ophthalmology, Tech4Health Institute & Neuroscience Institute, NYU Grossman School of Medicine, NYU Langone HealthNew York University 5894 New York NY 10016 USA; ^8^ Department of Biomedical Engineering, Tandon School of EngineeringNew York University 5894 New York NY 10016 USA; Spencer Center for Vision Research, Byers Eye Institute, Department of OphthalmologyStanford University School of Medicine 10624 Palo Alto CA 94303 USA

**Keywords:** Basic and instrumental activities of daily living, mobile technology, persons with blindness or low vision, semantic segmentation models, visual assistive technology

## Abstract

*Goal:* Persons with blindness or low vision (pBLV) face challenges in completing activities of daily living (ADLs/IADLs). Semantic segmentation techniques on smartphones, like DeepLabV3+, can quickly assist in identifying key objects, but their performance across different indoor settings and lighting conditions remains unclear. *Methods:* Using the MIT ADE20K SceneParse150 dataset, we trained and evaluated AI models for specific indoor scenes (kitchen, bedroom, bathroom, living room) and compared them with a generic indoor model. Performance was assessed using mean accuracy and intersection-over-union metrics. *Results:* Scene-specific models outperformed the generic model, particularly in identifying ADL/IADL objects. Models focusing on rooms with more unique objects showed the greatest improvements (bedroom, bathroom). Scene-specific models were also more resilient to low-light conditions. *Conclusions:* These findings highlight how using scene-specific models can boost key performance indicators for assisting pBLV across different functional environments. We suggest that a dynamic selection of the best-performing models on mobile technologies may better facilitate ADLs/IADLs for pBLV.

## I. Introduction

Basic and instrumental activities of daily living (ADLs, IADLs) – such as eating, cooking, toileting, bathing, and dressing - are essential for independent living. These tasks are often more challenging for persons with blindness or low vision (pBLV) and mostly occur indoors at home. Assistive technologies that leverage artificial intelligence (AI) can help, but there is an absence of AI models specifically trained to parse everyday environments and key objects that pBLV interact with to complete their daily tasks. This limits the functionality and practicality of assistive technologies to aid pBLV in completing their essential activities efficiently and safely, which negatively impacts their quality of life [1], [2]. With an aging population, and increasing prevalence of irreversible, age-related visual disorders [3], there is a growing unmet need to develop reliable approaches, tools, and AI that can facilitate visual rehabilitation for pBLV.

Advances in computer vision and machine learning techniques provide a promising basis for improving assistive technologies to aid pBLV in scene understanding, navigation, and human-environmental interaction. Semantic segmentation could provide a unique range of benefits due to its ability for pixel-level precision in classifying object identities within an image. While the potential for AI in assistive technology is widely recognized, most research focuses on object detection [4], which lacks spatial precision, or explores computationally intensive segmentation methods unsuitable for smartphones. To ensure that AI-assistance remains accessible to pBLV, utilizing smartphone-compatible methods for understanding the environment is important. Prior studies have compared the performance of a range of object detection and segmentation approaches on smartphones, including R-CNN, Fast R-CNN, Faster R-CNN, YOLO, Fast YOLOv2, Single-Shot Detection, and DeepLabV3+ in terms of accuracy and inference time, finding that Fast YOLOv2 (mAP 77.8; 59 FPS) and DeepLabV3+ (mAP 81.3; 44 FPS) were the best performing object detection and segmentation methods on mobile [5]. Furthermore, prototype smartphone applications that combine YOLO or DeepLabV3+ with distance estimation information have been developed in order to aid pBLV [5], [6].

This study advances this and related fields by focusing on optimizing smartphone-compatible semantic segmentation approaches for pBLV. Through discriminating the visuospatial distribution of objects in an image, semantic segmentation allows for more detailed forms of scene understanding and feedback for pBLV than other approaches, either by visually enhancing key objects, or controlling multimodal feedback (e.g., verbal descriptions, spatialized tones, vibrotactile alerts) to enhance accessibility for pBLV [7], [8]. The silhouettes it generates are also well-suited for sensory substitution through conveying object size, shape, and distance via patterns of sound or touch [9], [10], [11]. For example, sonifying images through converting pixel contents into loudness/timbre, pixel height into pitch, and pixel laterality into panning/time, can create visually meaningful soundscapes that preserve the location, size, shape, and identity of visual objects. As such, if an image contained a coffee table in the bottom left and TV in the top right, this could be heard as low-pitched wooden taps on the left, followed by high-pitched TV static on the right. This allows users to create an accurate mental reconstruction of the original image's contents, which can then be used to explore and act upon the visual world.

In this study, we implemented and evaluated the performance of 4 scene-specific semantic segmentation models (living room, kitchen, bedroom, bathroom) and 1 generic indoor model based on the smartphone-compatible DeepLabV3+ architecture [12]. Training was conducted on the MIT ADE20K SceneParse150 dataset, which includes a diverse range of annotated indoor scenes crucial for everyday activities within kitchens, bathrooms, bedrooms, and living rooms. Our goal is to measure the performance of generic indoor models and evaluate any alterations to performance from using scene-specific models within their respective rooms, including for segmenting key objects for ADLs/IADLs, and under varying image brightness conditions. These results can help determine the potential of selectively leveraging specific models for different room scenarios to provide better support for pBLV in completing ADLs/IADLs [13]. This study is unique in refining smartphone-compatible models for pBLV, making AI-powered visual rehabilitation tools more accessible on users' devices.

## II. Materials and Methods

### A. Background and Computer-Vision Model Selection

Computer vision techniques available on smartphones, such as the YOLO (“You Only Look Once”) object detection system [4] provide rapid object detection capabilities by generating a bounding box around an object, but lack the spatial precision needed for tasks involving precise object manipulation. Previously, we examined training and re-training these models to focus on, or add, key objects for pBLV [14], [15]. However, semantic segmentation more accurately preserves the shape, size, orientation, and spatial distribution of objects within an image, giving more information to the end user. This level of detail helps provide crucial information for many tasks, such as properly orienting the hand to grasp an object or orientating the body to navigate complex indoor spaces and obstacles more effectively and safely.

Smartphone-compatible semantic segmentation models like DeepLabV3+ enable pixel-wise segmentation via dilated convolutions and spatial pyramid pooling [12]. DeepLabV3+ is natively compatible with the iPhone, which is a common choice of smartphone for pBLV [16]. Apple's CoreML framework allows seamless integration of specific AI-models to iOS devices, with the ‘Semantic Segmentation-CoreML’ project showcasing that DeepLabV3+ currently provides real-time semantic segmentation for 20 object categories on iOS devices by default [17].

Leveraging DeepLabV3+ on smartphones enables more practical assistive technologies by combining precise object segmentation with distance data from sensors like Light Detection and Ranging (LiDAR) [10]. This type of integration should help in more accurately assigning distances to specific objects. However, as current CoreML DeepLabV3+ models on smartphones are limited to only 20 object categories, this highlights the need for custom training of these models to recognize more objects relevant to pBLV to expand their utility. Yet, to date, few studies have evaluated the impact of customizing the AI-model's training to refine their performance for tasks relevant to pBLV.

### B. Materials

In this study, we used the MIT ADE20K SceneParse150 dataset [18], which includes a diverse range of annotated scenes with more focuses on indoor scenarios. This dataset features high quality annotations and is the largest open-source dataset for semantic segmentation and scene parsing. It contains 22210 annotated images with 150 objects including 35 ‘stuff’ classes that represent amorphous background regions (e.g., wall, floor, sky) and 115 discrete object classes (e.g., table, sink, bed). This dataset is divided into 20k training images and 2k validation images, with each image having a scene category. The training and validation data splits were based on the standard ADE20K partition [18]. All images were unique with no repetitions - either across scenes or between the training and validation datasets. This allows us to use high volumes of image data and annotated masks based on different indoor scenes and provides a robust foundation for training and validating our models across different environments. This includes kitchens, bedrooms, living rooms, and bathrooms – spaces where pBLV can benefit from scene descriptions, navigation support, and object parsing.

For the generic indoor model, we used all indoor scene categories from the ADE20K dataset — in addition to the four scene-specific types. This included additional indoor environments such as dining rooms, pantries, and corridors. As a result, the generic indoor model was trained on a more diverse set of indoor scenes and featured additional object exemplars over the scene-specific models. This makes the generic indoor model a more comprehensive and representative baseline for indoor environments to evaluate any changes in performance for the scene-specific models. This comparison presents a more challenging test for the scene-specific models, with their superior performance highlighting their ability to specialize and outperform a more broadly trained model in their respective environments.

### C. Methods

To train pixel-level segmentation models for indoor scenes, we chose DeepLabV3+ as our basic semantic segmentation model to develop different scene-specific models and assess their segmentation accuracy of indoor scenes and objects. Details on the model training parameters can be found in the supplementary materials.

The ADE20K SceneParse150 dataset contains a variety of indoor and outdoor scenes. Since outdoor scenes such as “crossroad” or “vehicle” are irrelevant to the project goals, these scenes were excluded from our revised dataset. Instead, to achieve better performance and apply semantic segmentation models specific to indoor environments, we reviewed the ADE20K SceneParse150 dataset and identified 14 indoor scenes. To focus on the basic task requirements of pBLV, we selected the 4 most important scenes for daily tasks and curated specific training and validation datasets focused on the “kitchen”, “bedroom”, “bathroom”, and “living room” scenes. Furthermore, besides evaluating the accuracy of segment object pixels across these scenes, we also sought to evaluate the performance of these models in relatively dark environments. For this validation, images had their brightness decreased from their RGB values being multiplied by 1 to 0.1, in 0.1 decrements. As a result, we used validation images from these scenes, either unaltered, or with their brightness artificially lowered. Details on the distribution of training and validation image data used for each model can be found in the supplementary materials and in Supplementary Tables 2 to 6.

To compare each model's pixel segmentation capabilities, we selected 3 metrics commonly used for assessing semantic segmentation performance [16]: **Mean accuracy** indicates the proportion of correctly classified pixels averaged over all the classes (C) (1); **Mean IoU (mIoU)** indicates the intersection-over-union (IoU) between the predicted and ground-truth pixels, averaged across all the classes, with equal weight given to all classes present (2); **Weighted IoU (wIoU)** uses the IoU for each class present, but its contribution to the final wIoU score is weighted by the total pixel ratio of each class (i.e., visually smaller or less prevalent classes are weighted lower than larger or more prevalent classes). The total pixel ratio of each class refers to the proportion of pixels in an image (or across a dataset) that belong to that specific class (3).

\begin{align*}
&{\mathrm{Mean\ Accuracy}} = \frac{1}{\mathrm{C}} \sum_{\mathrm{i} = 1}^{\mathrm{C}} \frac{{{\mathrm{Correct\ Pixe}}{{\mathrm{l}}_{\mathrm{i}}}}}{{{\mathrm{Total\ Pixe}}{{\mathrm{l}}_{\mathrm{i}}}}} \tag{1}\\
&{\mathrm{mIoU\ }} = \ \frac{1}{C} \sum_{\mathrm{i} = 1}^{\mathrm{C}} \\
& \qquad\qquad \times\left( {\frac{{{\mathrm{True\ Positives\ }}{{{\left( {\text{TP}} \right)}}_{\mathrm{i}}}}}{{\mathrm{T}{{\mathrm{P}}_{\mathrm{i}}} \!+\! \ {{{\begin{array}{cccccc} {{\mathrm{False}}}\\ {{\mathrm{Positives}}\ \left( {{\mathrm{FP}}} \right)} \end{array}}}_{\mathrm{i}}} \!+\! {{{\begin{array}{cccccc} {{\mathrm{False}}\ }\\ {{\mathrm{Negatives}}\ \left( {{\mathrm{FN}}} \right)} \end{array}}}_{\mathrm{i}}}}}} \right) \tag{2}\\
&{\mathrm{wIoU\ }} = \ \sum_{\mathrm{i} = 1}^{\mathrm{C}} \left( {\left( {\frac{{\mathrm{T}{{\mathrm{P}}_{\mathrm{i}}}}}{{\mathrm{T}{{\mathrm{P}}_{\mathrm{i}}} + {\mathrm{\ F}}{{\mathrm{P}}_{\mathrm{i}}} + {\mathrm{\ F}}{{\mathrm{N}}_{\mathrm{i}}}}}} \right){\mathrm{*\ Pixel\ Rati}}{{\mathrm{o}}_{\mathrm{i}}}} \right) \tag{3}
\end{align*}

We describe details of how to use these three metrics to evaluate models in supplementary materials.

Finally, to specifically focus on key objects within these scenes, we also evaluated the mIoU for specific object classes (rather than overall) for the ‘top 5’ most important objects for ADLs and IADLs within each scene. This top 5 list was determined by consensus among experimenters as to which objects within the ADE20K dataset best relate to the primary ADLs/IADLs that occur within these rooms. For example, as the kitchen is used for cooking, eating, and cleaning dishes, the top 5 key objects are determined to be the dishwasher, cabinet, refrigerator, stove, and microwave.

## III. Results

**Fig. 1.**
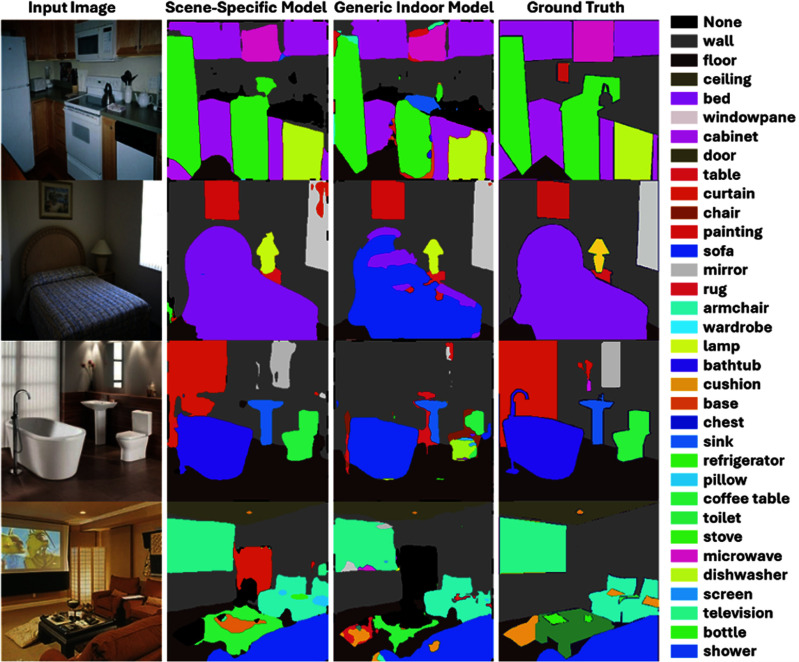
Examples of scene-specific and generic indoor model visualization on different room environments. Left column shows the input images. Middle-left and middle-right columns show the scene-specific and generic indoor model results, respectively, with colors indicating object identification category. Rightmost column shows ground-truth segmentations.

*Segmentation Performance:* Results of the scene-specific models and generic indoor model, in terms of mean accuracy, mIoU, and wIoU, across the four scenes are shown in Table I, while examples of model performance are provided in Fig. 1.

**TABLE I:** Scene-Specific vs Generic Indoor Model Performance on Different Room Environments (**BOLD +** Underlined = Best Performing in Scene)

**Scene**	Model type	Mean accuracy	mIoU (±S.D.)	wIoU (±w.S.D.)
Kitchen (67 trials)	Specific	**27.92%**	**0.234** ±0.218	**0.520** ±0.179
	Generic	26.69%	0.210 ±0.193	0.472 ±0.164
Bedroom (139 trials)	Specific	**34.65%**	**0.275 ±**0.228	**0.613** ±0.183
	Generic	30.77%	0.229 ±0.234	0.441 ±0.285
Bathroom (67 trials)	Specific	**43.11%**	**0.363 ±**0.240	**0.596** ±0.131
	Generic	29.10%	0.230 ±0.210	0.424 ±0.214
Living room (70 trials)	Specific	31.16%	0.244 ±0.218	**0.524** ±0.199
	Generic	**33.85%**	**0.269** ±0.219	0.522 ±0.204

The scene-specific model had a higher **mean accuracy** than the generic model in the kitchen (27.92% vs 26.69%), bedroom (34.65% vs 30.77%) and bathroom (43.11% vs 29.10%). However, the specific model performed worse than the generic model for the living room (31.16% vs 33.85%).

For **mIoU**, where evaluations of object segmentations are weighted evenly, scene-specific models had higher mIoU than generic models for the kitchen (0.2344 vs 0.2100), bedroom (0.2751 vs 0.2289), and bathroom (0.3628 vs 0.2302). By contrast, for the living room, the scene-specific model had a lower mIoU than the generic model (0.2444 vs 0.2686).

For **wIoU**, where evaluations of object segmentations are weighted according to the proportion of pixels they occupy, the scene-specific model had a higher wIoU than the generic model for all scenes, with kitchen (0.5204 vs 0.4721), bedroom (0.6134 vs 0.4410), bathroom (0.5957 vs 0.4236), and living room (0.5242 vs 0.5220).

*Key object performance:* To evaluate each model's ability to facilitate daily tasks, we compared scene-specific and generic models on the top 5 most relevant objects for ADLs/IADLs in each room in terms of mIoU and mean accuracy for specific objects (see Table II).

**TABLE II:** Segementation Model Performance for Scene-Specific Key Objects (**BOLD +** Underlined = Best Performing in Key Objects)

		Scene-specific model		Generic indoor model	
**Scene**	Key objects	Class mIoU (±S.D.)	Class mean accuracy	Class mIoU (±S.D.)	Class mean accuracy
Kitchen (67 trials)	Dishwasher	**0.64** ±0.42	**0.683**	0.26 ±0.29	0.266
	Cabinet	**0.62** ±0.23	**0.796**	0.53 ±0.24	0.611
	Fridge	**0.60** ±0.31	**0.695**	0.51 ±0.36	0.527
	Stove	**0.55** ±0.32	**0.607**	0.36 ±0.30	0.390
	Microwave	**0.33** ±0.36	**0.344**	0.21 ±0.33	0.211
Bed Room (139 trials)	Bed	**0.68** ±0.29	**0.910**	0.05 ±0.08	0.051
	Lamp	**0.51** ±0.23	**0.780**	0.49 ±0.26	0.643
	Chest	**0.50** ±0.34	**0.618**	0.23 ±0.27	0.236
	Wardrobe	**0.40** ±0.32	**0.432**	0.09 ±0.17	0.087
	Pillow	**0.36** ±0.26	**0.426**	0.04 ±0.10	0.039
Bath Room (67 trials)	Mirror	**0.61** ±0.28	**0.701**	0.14 ±0.26	0.144
	Bathtub	**0.64** ±0.34	**0.733**	0.17 ±0.15	0.170
	Sink	**0.60** ±0.32	**0.668**	0.33 ±0.27	0.352
	Toilet	**0.63** ±0.30	**0.709**	0.11 ±0.11	0.112
	Towel	**0.38** ±0.31	**0.507**	0.02 ±0.05	0.021
Living room (70 trials)	Sofa	**0.57** ±0.25	**0.819**	0.56 ±0.26	0.787
	Coffee table	**0.47** ±0.26	**0.541**	0.26 ±0.26	0.275
	TV	**0.53** ±0.33	**0.595**	0.45 ±0.37	0.506
	Cushion	**0.29** ±0.25	**0.329**	0.22 ±0.24	0.258
	Curtain	0.27 ±0.25	**0.776**	**0.49** ±0.30	0.642

We found that scene-specific models had higher mIoU for 19/20 classes and higher mean accuracy for 20/20 classes relative to generic models. This includes substantially higher mIoUs for objects such as the dishwasher (0.6409 vs 0.2562), stove (0.5475 vs 0.3574), bed (0.6841 vs 0.0504), toilet (0.6323 vs 0.1106), sink (0.6007 vs 0.3254), towel (0.3775 vs 0.0206), and coffee table (0.4723 vs 0.2615).

**Fig. 2.**
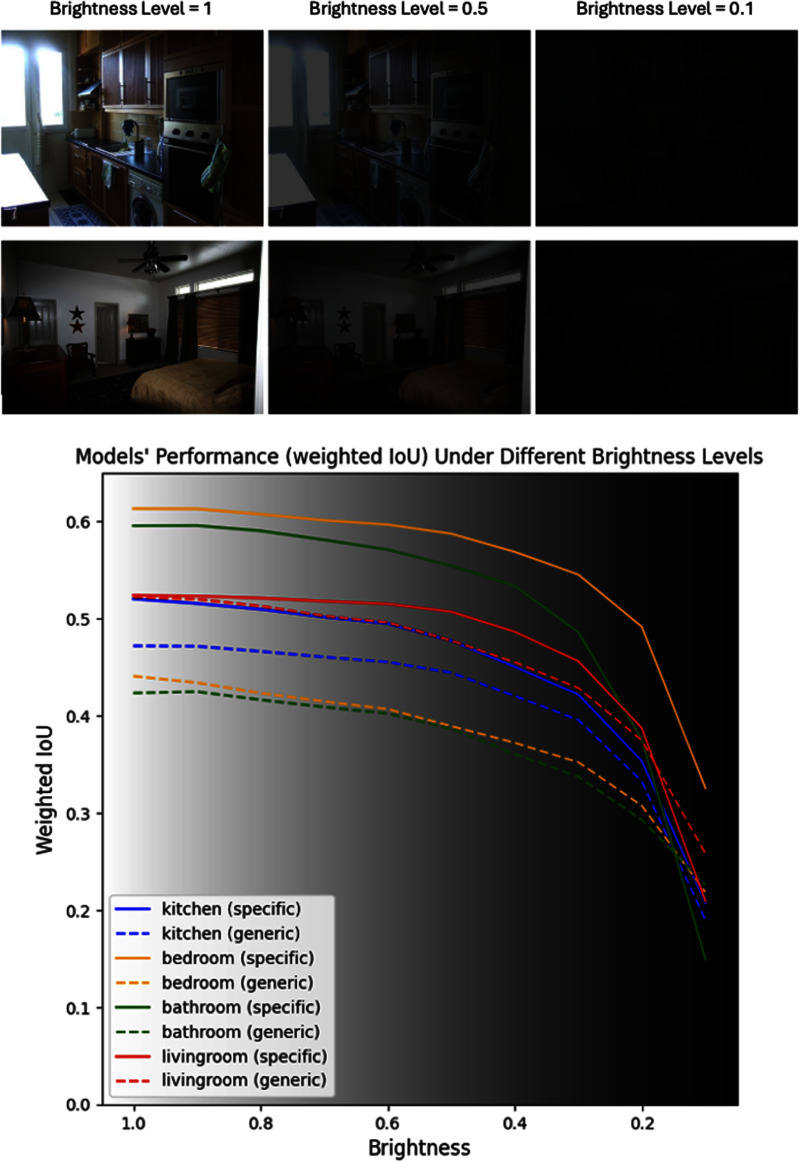
The top image illustrates **scene images under different brightness levels via multiplying RGB values by 1 (left column), 0.5 (middle column), or 0.1 (right column).** The bottom image shows **scene-specific vs generic indoor model performance (in wIoU) under different brightness levels.** The brightness level of 1 means an image has original brightness. The brightness level of 0.1 means an image has 10% of RGB values of the original image.

*Resilience to low brightness:* We assessed wIoU across images varying in brightness. All models had wIoU reducing with lower brightness. However, scene-specific models had higher starting wIoU values, and most scene-specific models had lower performance drops as a proportion of starting performance than generic models (e.g., living room, bedroom) across large reductions in brightness (see Fig. 2), suggesting resilience in dim lighting conditions.

Additional statistics evaluating segmentation metrics for each object across all models as well as proportion performance deterioration from reducing image brightness can be found in the supplemental materials.

## IV. Discussion

Our results show that scene-specific models outperform generic models in semantically segmenting objects in 3 out of 4 indoor spaces. Here the degree of improvement appeared to relate to the uniqueness of objects to each room type. Scene-specific models performed better in segmenting key objects for activities of daily living within each room. Finally, scene-specific models better maintained their superior performance even as the brightness of an image was reduced, demonstrating more robust performance under variations in lighting, which may aid real-world use where environmental lighting can be unpredictable and challenging. Here we discuss the reasons for, and implications of, these results as well as the utility of these models within the context of assisting daily tasks for pBLV.

The study uncovers three main findings relating to the model performance in specific scenes for different objects, and the overall model performance across brightness levels. First, the study shows that scene-specific models demonstrated superior performance in accurately segmenting specific rooms over generic models across three evaluation metrics (pixel accuracy, mIoU, wIoU). This was particularly evident in environments like bedrooms and bathrooms, which may be attributable to the uniqueness of the objects (e.g., beds, bathtubs) and the layout of these room types relative to other indoor environments. The scene-specific models may have enhanced their performance through avoiding misidentifying key objects as other visually similar objects (e.g., bath, sink). Furthermore, training models only on typical exemplars of a specific room-type may allow models to use the more predictable arrangement of objects in the training data to better fine-tune their segmentations.

Secondly, our data revealed that scene-specific models performed better with key objects in their respective rooms. For instance, in bedrooms, the scene-specific model substantially outperformed the generic indoor model in identifying beds with 13 times better mIoU and 18 times better mean accuracy. This highlights a critical weakness of the generic indoor model, where it often misidentified room-unique objects such as beds, instead as sofas or other visually similar objects more common in other rooms. This reveals a key limitation of generic models in over-applying a wide range of alternative object classes in a manner that reduces their accuracy and practicality for assistive technologies. Such misidentifications underscore the benefits of using scene-specific models within the correct context to enhance the performance and reliability of these technologies.

Thirdly, scene-specific models maintain better performance in mid- or even low-light conditions compared to the generic model. This adaptability is crucial because prior interviews with pBLV often describe that any remaining visual functionality is further impaired in low light conditions [19], and fully blind users may be less aware of a room's current lighting, and less aware of how sub-optimal lighting may negatively influence assistive technology performance. Furthermore, lighting may not always be controllable, especially in shared or public spaces [20], and so computer vision approaches that operate more consistently across a variety of lighting conditions will result in more reliable assistive technologies for end users.

It is important to consider that the images assessed here are of room-level overviews of the environment. As such, many key objects may have a small visual size. However, as the assistive technology user searches the room or approaches key objects, these visual sizes and details will increase relative to the room-level overview. Future investigations should seek to evaluate segmentation performance during these closer scenes and active use or exploration by potential end users to ensure robustness. It should be noted that key metrics such as wIoU are sensitive to object scale, and so wIoU performance for image segmentation may change substantially as different objects expand in the frame. To help account for this variation, we complemented wIoU scores with mIoU scores, which assign all object classes an equal weight, irrespective of their visual size, and so better reflect performance on visually small items. Furthermore, we expanded this group evaluation by reporting the mIoU scores for individual key objects of both larger (e.g., bed) and smaller (e.g., lamp) sizes. All of these metrics help provide a more balanced view of segmentation quality across both full scenes and individual objects important for daily tasks.

The overall mIoU scores for rooms averaged at 0.28 for the scene-specific model, and 0.23 for the generic indoor model. These scores are lower than the average overall wIoU scores (0.56 and 0.46 respectively), which is likely the result of mIoU scores being brought down by poor performance on rarer object categories. However, for the top 5 key objects in each room, mIoU is higher, averaging at 0.51 for the scene-specific model, and 0.27 for the generic indoor model. While not perfect, an IoU of 51% (or even 27%) is usable, because detecting the presence and location of objects provides actionable information for the user even if size and shape information is less accurate. Furthermore, some lower scores are the result of misrecognitions with visually or categorically similar objects, such as misrecognizing beds as couches in the bedroom, which lowers the mIoU rating but would still be usable by the end user.

Model performance under low-lighting conditions could also be further improved by refining the image processing pipeline and model. For instance, one prior study tested DeepLabV3+ with combination of additional dilated convolution layers with customized dilation rates, non-linear group normalization shortcuts, and geometrically bunched pixel cues, which produced marginal gains in IoU in the context of dark driving conditions [21]. Another approach could be to expand data augmentation methods to enhance AI training as well as integrating more sensor inputs to mitigate the effects of reduced image quality from low-light conditions. Software-based image enhancement methods could also denoise images as well as adjust brightness/exposure and contrast in real-time, prior to AI-processing, which could improve model accuracy in dim environments. Finally, activating external light sources, such as smartphone flashlights when necessary, may also offer practical solutions to mitigate the effects of reduced image quality.

Despite the generally higher performance of room-specific models, the generic model showcased higher performance in the living room environment. This may be because living rooms feature more room-agnostic object classes than other indoor spaces (e.g., chair, table). As a result, the generic model may be more effective in environments with common or misplaced objects, where broader recognition is beneficial.

Several implementation insights are suggested from these findings. The better performance of scene-specific models for specific rooms suggests that a dynamic, model-switching mechanism could maximize these benefits, by switching to the best performing model for the current environment in visual assistive devices. Model switching could be triggered manually, based on current tasks, or by the identification of scene-specific objects, either by generic DeepLabV3+ models or visual language models to identify the most likely room type (e.g., VisPercep [22]). By dynamically switching between models, this approach could adapt in real-time to changing environments, ensuring best performance available across different settings and thus better supporting the diverse needs of pBLV.

Future research should aim to refine semantic segmentation models and improve how they are selected for different environments to better support pBLV in daily life. Enhancing data augmentation, incorporating sensor inputs, and exploring additional AI models could address current limitations. Continued efforts to optimize these models for real-time use on portable devices is key for practical, impactful deployment.

## V. Conclusion

Here, we demonstrated a range of benefits attributable to using scene-specific models over generic indoor models for the semantic segmentation of common indoor scenes within home environments including kitchens, bedrooms, bathrooms, and living rooms. We found that the AI model performance was greater for rooms that featured more unique room-specific items (e.g., toilets in bathrooms) in terms of higher accuracy, as well as higher mean or weighted IoU. Scene-specific models also had improved segmentation for key objects relevant to daily tasks and a higher resistance to performance deterioration resulting from images with low brightness levels. These metrics can be important for users who have poor spatial or acuity contrast, where poor lighting can reduce the visual contrast level needed to perceive key objects or hazards. Our research advances the field of assistive technology by demonstrating the unique benefits of scene-specific models for pBLV, including improved overall accuracy, resilience to low-light conditions, and enhanced segmentation of key objects for daily living. This novel approach provides a practical and accessible pathway to empower pBLV in their daily lives by leveraging smartphone-compatible AI models in a more focused manner. By appropriately selecting scene-specific models for their unique room or ADL/IADL context, the functionality of visual assistive technologies for pBLV can be improved, ultimately with a view to enhancing their visual rehabilitation outcomes.

## Supplementary Materials

We provided more methodological details and supplementary tables of the counts and the proportion of each object class in each model. We also provided additional statistical comparisons of the individual model performance under different brightness conditions.

Supplementary Materials

## Authors Contribution

Ruijie Sun: Methodology, Software, Investigation, Data curation, Writing – original draft, Writing –review & editing, Visualization. Giles Hamilton-Fletcher: Conceptualization, Validation, Formal analysis, Writing – original draft, Writing – review & editing. Sahil Faizal: Software, Investigation, Writing – original draft. Chen Feng: Writing – review & editing. Todd Hudson: Writing – review & editing. John-Ross Rizzo: Writing – review & editing. Kevin Chan: Conceptualization, Writing – review & editing, Supervision, Funding.

## Conflict of Interest Statement

The authors declare that they have no conflict of interest.
