# Supplementary material for: Training Indoor and Scene-Specific Semantic Segmentation Models to Assist Blind and Low Vision Users in Activities of Daily Living
Source: IEEE Open J Eng Med Biol. 2025 Sep 9;6:533–9. doi: 10.1109/OJEMB.2025.3607816 (PMC12599903; doi:10.1109/OJEMB.2025.3607816)
Supplement: Supplementary Materials [file supp1-3607816.pdf]

Supplementary Materials

Training Indoor and Scene-Specific Semantic Segmentation Models to Assist Blind and Low Vision Users in Activities of Daily Living

Ruijie Sun, Giles Hamilton-Fletcher, Sahil Faizal, Chen Feng, Todd E. Hudson, John-Ross Rizzo, Kevin C. Chan*

I. Materials and Methods

Details of datasets. We used the training and validation splits provided by the MIT ADE20K SceneParse150 dataset, which is the same standard split used in the Scene Parsing Challenge. Importantly, each image in ADE20K is of a unique physical environment — there are no repeated frames, environments, or near-duplicates between the training and validation sets. Thus, the training and validation sets contain different kitchen, bathroom, bedroom, and living room scenes, allowing us to evaluate the generalization ability of scene-specific models to unseen examples of the same room type. We did not apply data augmentation during training, ensuring that no synthetic overlap was introduced between sets. In terms of data counts (RGB image + segmentation data), the 'kitchen' dataset has 661 training data as well as 67 validation data which provided the 67 evaluation trials to assess the model after each epoch and establish the mean accuracy, mIoU, and wIoU metrics for the fully-trained model; similarly, the 'bedroom' dataset has 1,389 training data and 139 validation data / evaluation trials; the 'bathroom' dataset has 671 training data and 67 validation data / evaluation trials; and the 'living room' dataset has 697 training data with 70 validation data / evaluation trials. The validation data was only used for testing trials in this study, and was not used for model training or hyperparameter tuning.

Training Performance. Models were trained for 60 epochs on a V100 GPU. For the scene-specific models, the training time of each epoch was 8, 17, 8, and 9 seconds for kitchen, bedroom, bathroom, and living room models respectively. The generic indoor model took 42 seconds per epoch (22.2% slower than the sum of the scene-specific models), this is because it contained all indoor categories, including those beyond the rooms used for the scene-specific models.

Supplementary Tables 1 to 5 show the counts and the proportion of each object class in each model using the MIT ADE20K SceneParse150 dataset. Although there is an imbalance in prevalence (e.g., "wall" class occupies 7.9% of object instances and "sofa, couch" class only occupies 0.3% of object instances in the kitchen dataset), important objects in a kitchen scene do appear at high frequencies. For example, 7.6% of object instances are "cabinet" class and 5.5% of object instances are "stove" class. In addition, we created a dataset containing all home indoor scenes to train a generic indoor model as a baseline for comparison. The generic indoor dataset has 4,360 training data and 441 validation data. In real-world scenarios, varying indoor lighting conditions are important to consider, especially for the fully blind users, where the status of environmental lighting may be hard to ascertain. It is also important to provide practical solutions for assistance in dim conditions for those with night blindness.

Model Training Details. We employed transfer learning by initializing the encoder of the DeepLabV3+ architecture with a ResNet50 backbone pretrained on ImageNet (1000 classes), allowing the model to leverage visual features learned from large-scale natural image data. All layers of the ResNet50 backbone were fine-tuned with no layers being frozen, enabling full adaptation to indoor scene characteristics. The Atrous Spatial Pyramid Pooling (ASPP) module included parallel convolutions with dilation rates of 1, 6, 12, and 18, along with global average pooling. The outputs were concatenated and passed through a 1×1 convolution. This architecture was used consistently across both generic indoor and scene-specific models.

Training was conducted for up to 60 epochs with early stopping (patience = 10), based on validation loss. We used the Adam optimizer with a fixed learning rate of 0.0005, and the loss function was sparse categorical cross-entropy. A batch size of 8 and image resolution of 256×256 pixels were used across all experiments, determined through hyperparameter tuning via random search.

Training was performed using Google Colab Pro using a V100 GPU at a cost of $0.49 per hour to cover the ~5 compute units used during that time period. All models were trained for 60 epochs with one additional run to test the trained model to predict the testing set once. Here the kitchen, bedroom, bathroom, living room, and indoor models took 8, 17, 8, 9, and 54 seconds per epoch respectively, which results in a model training cost of $0.07, $0.14, $0.07, $0.07, and $0.44 respectively. The python script used for model training is available upon request.

How to use the performance metrics. The IoU metric measures the overlap between the predicted segmentation and the ground truth for each class, offering a clear indication of the model's performance in delineating the spatial distribution of different objects within an image. However, there is an imbalanced distribution of objects, both for the number of pixels associated with that class in individual images, and in their prevalence across all images within a specific scene. Metrics such as mIoU do not account for this imbalance and weight all object classes evenly. For example, if there are 100 classes, the IoU for each class will contribute 1% to the final

mIoU score. However, some object classes may be more prevalent or may contribute to a larger number of pixels within the images than other object classes. The wIoU weights the contribution of each class to the final wIoU score according to the proportion of pixels associated with that class across all images within that scene. For example, if the "bed" object class takes up 10% of pixels in the ground-truth bedroom images, then the IoU score for the "bed" object class would contribute to 10% of the final wIoU score. Overall, mIoU may have an outsized influence of small or rare objects on its final score, while wIoU will balance accordingly but the scores may become influenced by background regions (e.g. walls, floors, ceilings) that take up a large proportion of the scene.

As a result, we used mIoU to evaluate segmentation performance for specific key objects, as this metric focuses on a specific object class and summarizes the IoU performance across all images where the object is present in the ground truth data and/or predicted by the model. The mIoU approach gives equal weight for each object class, irrespective of the object's visual size or rarity across images, which provides an intuitive and comparative summary of model performance for each object. The mIoU highlights how well the model segments each object of interest in isolation, which is most relevant for assessing assistive capabilities for ADLs/IADLs. By contrast, we used wIoU to evaluate model performance under varying brightness conditions. This is because wIoU accounts for each class's total contribution in terms of pixels across all images, with the resulting score better reflecting the overall segmentation quality of the full image. This is important when assessing general model robustness and usability across full scenes. Both mIoU and wIoU have their distinct advantages and disadvantages. However, taken together, they provide a more holistic evaluation of each model's performance.

II. Results

More in-depth statistical assessments about reducing image brightness. To evaluate the segmentation model performance under different brightness conditions, we assessed wIoU across varying image brightness levels and illustrated a trend of diminishing wIoU with decreasing brightness. Fig. 2 in the paper showed that in general, both scene-specific and generic indoor models initially exhibited a stable performance when the lighting condition was only 50% of the original image, but the performance dropped more substantially when the brightness level reached below 0.5, indicating that these models were able to continue similar performance in mildly dim environments.

In terms of individual model performance, while all models performed worse as the brightness levels decreased in the input image, this deterioration occurred at different rates for different models, irrespective of their overall performance levels at 100% brightness. This indicates that certain models are more resistant to changes in lighting levels than others. To illustrate this point, we showcased the model performance loss from fully bright images to 50% and 40% brightness levels (Supplementary Table 1).

SUPPLEMENTARY TABLE 1.
SEGEMENTATION MODEL PERFORMANCE CHANGES UNDER DIFFERENT BRIGHTNESS LEVELS (**BOLD** = BEST PERFORMING IN SCENE)

**Scene**	**wIoU performance change relative to 1.0 brightness**	
	0.5 brightness	0.4 brightness
Kitchen (specific)	-8.26%	-13.45%
Kitchen (generic)	**-5.85%**	**-10.91%**
Bedroom (specific)	**-4.22%**	**-7.25%**
Bedroom (generic)	-11.68%	-15.54%
Bathroom (specific)	**-6.92%**	**-10.36%**
Bathroom (generic)	-8.69%	-14.81%
Living room (specific)	**-3.21%**	**-7.15%**
Living room (generic)	-8.54%	-12.78%

We observed that all scene-specific models demonstrated a smaller drop in performance relative to the generic indoor model, with the exception of the kitchen scene. In the kitchen, the scene-specific model's wIoU decreased by 8.26% at 50% brightness, which is greater than the 5.85% decrease observed in the generic model. For the bedroom, the specific model exhibited a smaller reduction in performance compared to the generic model. At 50% brightness, the scene-specific model's wIoU decreased by 4.22%, which is less than the generic model's 11.68% drop. In the bathroom, the scene-specific model's wIoU dropped by 6.92% at 50% brightness, whereas the generic model had a larger decline of 8.69%. Finally, the living room scene highlighted the robustness of the scene-specific model under reduced lighting conditions, with the scene-specific model's wIoU decreased by only 3.21% at 50% brightness compared to the more substantial reduction in the generic model by 8.54%. These trends continued at 40% brightness, where the differences in performance drop widened further between the scene-specific model and generic model. Overall, this data shows that there are differing levels of resistance to reducing image brightness values according to both the type of model (i.e., generic, scene-specific) as well as the indoor room type (i.e., kitchen, bedroom, bathroom, living room).

III. Discussion

Future Model Enhancements. While we show a novel solution for ensuring more consistent performance across unpredictable lighting scenarios, future research could also expand how models in low-light conditions are assessed or have issues mitigated through several methods. In real-world scenarios, the cameras used in smartphones or other assistive technologies typically introduce additional image artifacts in low-light conditions that are not accounted for in the present analysis. These include, but are not limited to, additional visual noise, blur, more limited dynamic ranges, as well as reduced

contrast, sharpness, and color saturation or accuracy. While our data highlights the effect of lowering image brightness without these effects on model performance, future investigations should also look into the effects of degraded image quality on model performance. This could be done either by adding accurately modeled forms of degraded visual images based on realistic sensor profiles, or by creating new datasets under controlled lighting conditions.

Explaining Model Enhancements. We observed that while the room-specific models tended to have higher performance for the majority of rooms, the generic model showcased higher performance in the living room environment. This could be explained by the generic model being able to leverage additional training data from other scenes to better segment objects that are present in living rooms but also present in other scenes. This suggests less impact for scene-specific training in environments that share more object classes with other rooms as opposed to the more unique room types. Another potential benefit for the generic model may involve scenarios where a user encounters an object that is misplaced or typically not fixed to a specific room, such as a cup or a towel. Here, the generic indoor model could provide broader recognition capabilities, making it more effective for unexpected objects or mixed environments.

SUPPLEMENTARY TABLE 2 – GENERIC INDOOR MODEL

Class Name	Count	Percentage	Class Name	Count	Percentage	Class Name	Count	Percentage
wall	4325	8.341	ottoman	240	0.463	rock	7	0.013
floor	3991	7.696	dishwasher	229	0.442	sea	6	0.012
ceiling	2852	5.5	stool	203	0.391	flag	6	0.012
table	2443	4.711	oven	199	0.384	fence	6	0.012
windowpane	2416	4.659	blanket	150	0.289	mountain	5	0.01
lamp	2024	3.903	kitchen island	142	0.274	case	5	0.01
painting	1978	3.814	screen door	131	0.253	boat	5	0.01
cabinet	1899	3.662	railing	130	0.251	animal	5	0.01
chair	1460	2.816	desk	124	0.239	swimming pool	3	0.006
door	1449	2.794	radiator	113	0.218	arcade machine	3	0.006
curtain	1427	2.752	column	113	0.218	pole	2	0.004
bed	1383	2.667	shower	104	0.201	palm	2	0.004
light	1160	2.237	swivel chair	98	0.189	fountain	2	0.004
cushion	1132	2.183	pool table	98	0.189	booth	2	0.004
plant	998	1.925	buffet	97	0.187	bicycle	2	0.004
sink	994	1.917	bookcase	93	0.179	awning	2	0.004
mirror	904	1.743	person	92	0.177	trade name	1	0.002
vase	903	1.741	plaything	85	0.164	tower	1	0.002
rug	815	1.572	step	83	0.16	tank	1	0.002
sofa	812	1.566	ashcan	77	0.148	streetlight	1	0.002
flower	757	1.46	bag	71	0.137	land	1	0.002
armchair	743	1.433	sculpture	70	0.135	car	1	0.002
pillow	717	1.383	signboard	60	0.116	airplane	1	0.002
shelf	593	1.144	poster	58	0.112	waterfall	0	0
coffee table	570	1.099	counter	58	0.112	water	0	0
book	569	1.097	apparel	57	0.11	van	0	0
box	550	1.061	bench	51	0.098	truck	0	0
pot	538	1.038	tree	50	0.096	traffic light	0	0
sconce	511	0.985	stairway	45	0.087	tent	0	0
bottle	482	0.93	stairs	42	0.081	stage	0	0
stove	476	0.918	sky	33	0.064	skyscraper	0	0
chest of drawers	440	0.849	crt screen	31	0.06	sidewalk	0	0
towel	417	0.804	food	30	0.058	ship	0	0
fireplace	391	0.754	seat	28	0.054	sand	0	0
bathtub	384	0.741	canopy	27	0.052	runway	0	0
plate	383	0.739	bannister	24	0.046	road	0	0
television	379	0.731	screen	23	0.044	river	0	0
chandelier	375	0.723	ball	23	0.044	pier	0	0
toilet	368	0.71	washer	19	0.037	path	0	0
clock	355	0.685	monitor	17	0.033	minibike	0	0
basket	355	0.685	computer	16	0.031	lake	0	0
refrigerator	318	0.613	bar	16	0.031	hovel	0	0
tray	301	0.58	escalator	13	0.025	hill	0	0
glass	301	0.58	base	13	0.025	grandstand	0	0
countertop	299	0.577	building	10	0.019	field	0	0
wardrobe	280	0.54	grass	9	0.017	dirt track	0	0
microwave	280	0.54	cradle	9	0.017	conveyer belt	0	0
fan	266	0.513	house	8	0.015	bus	0	0
hood	251	0.484	earth	8	0.015	bridge	0	0
blind	243	0.469	bulletin board	8	0.015	barrel	0	0

SUPPLEMENTARY TABLE 3 – KITCHEN MODEL

Class Name	Count	Percentage	Class Name	Count	Percentage	Class Name	Count	Percentage
wall	660	7.864	washer	13	0.155	swimming pool	0	0
cabinet	634	7.554	mirror	10	0.119	streetlight	0	0
floor	580	6.911	bag	10	0.119	stage	0	0
ceiling	466	5.552	wardrobe	9	0.107	skyscraper	0	0
stove	463	5.517	plaything	9	0.107	sidewalk	0	0
sink	451	5.374	radiator	8	0.095	shower	0	0
windowpane	387	4.611	poster	8	0.095	ship	0	0
refrigerator	295	3.515	countertop	8	0.095	sea	0	0
light	272	3.241	sculpture	7	0.083	screen	0	0
microwave	265	3.157	bench	7	0.083	screen door	0	0
hood	238	2.836	bed	7	0.083	sand	0	0
chair	237	2.824	crt screen	6	0.071	runway	0	0
dishwasher	228	2.717	signboard	5	0.06	road	0	0
table	227	2.705	fireplace	5	0.06	river	0	0
door	207	2.466	coffee table	5	0.06	pool table	0	0
bottle	188	2.24	step	4	0.048	pole	0	0
oven	187	2.228	bookcase	4	0.048	pier	0	0
lamp	178	2.121	blanket	4	0.048	path	0	0
plant	171	2.037	stairs	3	0.036	palm	0	0
chest of drawers	140	1.668	buffet	3	0.036	mountain	0	0
plate	131	1.561	tree	2	0.024	minibike	0	0
kitchen island	130	1.549	swivel chair	2	0.024	land	0	0
painting	129	1.537	seat	2	0.024	lake	0	0
tray	107	1.275	railing	2	0.024	hovel	0	0
vase	104	1.239	ottoman	2	0.024	hill	0	0
shelf	102	1.215	house	2	0.024	grass	0	0
basket	100	1.191	desk	2	0.024	grandstand	0	0
curtain	93	1.108	bar	2	0.024	fountain	0	0
flower	92	1.096	bannister	2	0.024	flag	0	0
pot	91	1.084	ball	2	0.024	field	0	0
rug	78	0.929	toilet	1	0.012	fence	0	0
box	69	0.822	stairway	1	0.012	escalator	0	0
book	65	0.774	sky	1	0.012	earth	0	0
glass	64	0.763	rock	1	0.012	dirt track	0	0
stool	60	0.715	pillow	1	0.012	cradle	0	0
clock	43	0.512	monitor	1	0.012	conveyer belt	0	0
chandelier	33	0.393	computer	1	0.012	car	0	0
blind	32	0.381	case	1	0.012	canopy	0	0
sofa	28	0.334	bulletin board	1	0.012	bus	0	0
counter	27	0.322	bathtub	1	0.012	building	0	0
food	25	0.298	base	1	0.012	bridge	0	0
television receiver	19	0.226	waterfall	0	0	booth	0	0
fan	19	0.226	water	0	0	boat	0	0
ashcan	18	0.214	van	0	0	bicycle	0	0
towel	17	0.203	truck	0	0	barrel	0	0
person	17	0.203	traffic light	0	0	awning	0	0
column	17	0.203	trade name	0	0	arcade machine	0	0
cushion	15	0.179	tower	0	0	apparel	0	0
sconce	14	0.167	tent	0	0	animal	0	0
armchair	14	0.167	tank	0	0	airplane	0	0

SUPPLEMENTARY TABLE 4 – BATHROOM MODEL

Class Name	Count	Percentage	Class Name	Count	Percentage	Class Name	Count	Percentage
wall	671	9.966	signboard	4	0.059	pool table	0	0
floor	568	8.436	sculpture	4	0.059	pole	0	0
sink	523	7.768	clock	4	0.059	pier	0	0
mirror	409	6.075	bench	4	0.059	path	0	0
bathtub	371	5.51	bannister	4	0.059	palm	0	0
cabinet	370	5.495	tree	3	0.045	oven	0	0
towel	363	5.391	ottoman	3	0.045	monitor	0	0
toilet	359	5.332	fireplace	3	0.045	minibike	0	0
ceiling	292	4.337	armchair	3	0.045	land	0	0
countertop	282	4.188	sofa	2	0.03	lake	0	0
windowpane	258	3.832	plaything	2	0.03	kitchen island	0	0
door	232	3.446	pillow	2	0.03	hovel	0	0
curtain	200	2.97	fan	2	0.03	house	0	0
sconce	146	2.168	cushion	2	0.03	hood	0	0
shelf	130	1.931	tower	1	0.015	hill	0	0
screen door	127	1.886	sky	1	0.015	grass	0	0
bottle	125	1.857	refrigerator	1	0.015	grandstand	0	0
box	112	1.663	poster	1	0.015	fountain	0	0
shower	102	1.515	person	1	0.015	food	0	0
vase	101	1.5	mountain	1	0.015	flag	0	0
light	100	1.485	microwave	1	0.015	field	0	0
painting	99	1.47	earth	1	0.015	fence	0	0
rug	89	1.322	coffee table	1	0.015	escalator	0	0
plant	81	1.203	ball	1	0.015	dishwasher	0	0
flower	80	1.188	apparel	1	0.015	dirt track	0	0
glass	66	0.98	waterfall	0	0	desk	0	0
basket	59	0.876	water	0	0	crt screen	0	0
chair	39	0.579	van	0	0	cradle	0	0
pot	37	0.55	truck	0	0	conveyer belt	0	0
step	34	0.505	traffic light	0	0	computer	0	0
blind	26	0.386	trade name	0	0	case	0	0
ashcan	26	0.386	tent	0	0	car	0	0
railing	25	0.371	tank	0	0	canopy	0	0
table	21	0.312	swivel chair	0	0	bus	0	0
radiator	21	0.312	swimming pool	0	0	bulletin board	0	0
tray	18	0.267	streetlight	0	0	building	0	0
lamp	16	0.238	stove	0	0	buffet	0	0
stool	13	0.193	stairway	0	0	bridge	0	0
bag	13	0.193	stage	0	0	booth	0	0
plate	10	0.149	skyscraper	0	0	bookcase	0	0
chandelier	10	0.149	sidewalk	0	0	boat	0	0
chest of drawers	9	0.134	ship	0	0	blanket	0	0
wardrobe	8	0.119	seat	0	0	bicycle	0	0
counter	8	0.119	sea	0	0	base	0	0
stairs	7	0.104	screen	0	0	barrel	0	0
column	6	0.089	sand	0	0	bar	0	0
book	5	0.074	runway	0	0	awning	0	0
bed	5	0.074	rock	0	0	arcade machine	0	0
washer	4	0.059	road	0	0	animal	0	0
television receiver	4	0.059	river	0	0	airplane	0	0

SUPPLEMENTARY TABLE 5 - LIVING ROOM MODEL

Class Name	Count	Percentage	Class Name	Count	Percentage	Class Name	Count	Percentage
wall	693	6.013	stairs	14	0.121	traffic light	0	0
floor	681	5.909	radiator	13	0.113	trade name	0	0
sofa	622	5.397	swivel chair	11	0.095	tower	0	0
ceiling	562	4.876	sky	10	0.087	tent	0	0
lamp	538	4.668	buffet	10	0.087	tank	0	0
table	530	4.599	bed	10	0.087	swimming pool	0	0
painting	530	4.599	plaything	9	0.078	streetlight	0	0
windowpane	504	4.373	kitchen island	8	0.069	stage	0	0
cushion	500	4.338	ball	8	0.069	skyscraper	0	0
coffee table	469	4.069	refrigerator	7	0.061	signboard	0	0
armchair	433	3.757	crt screen	7	0.061	sidewalk	0	0
plant	343	2.976	poster	6	0.052	shower	0	0
cabinet	342	2.967	person	6	0.052	ship	0	0
rug	326	2.829	hood	6	0.052	screen	0	0
vase	307	2.664	bench	6	0.052	screen door	0	0
chair	302	2.62	bar	6	0.052	sand	0	0
light	290	2.516	bag	6	0.052	runway	0	0
fireplace	279	2.421	oven	5	0.043	road	0	0
curtain	275	2.386	microwave	5	0.043	river	0	0
door	271	2.351	chest of drawers	5	0.043	pole	0	0
book	251	2.178	seat	4	0.035	pier	0	0
pot	220	1.909	counter	4	0.035	path	0	0
flower	211	1.831	computer	4	0.035	monitor	0	0
television receiver	196	1.701	stove	3	0.026	minibike	0	0
shelf	148	1.284	rock	3	0.026	land	0	0
mirror	139	1.206	boat	3	0.026	lake	0	0
ottoman	138	1.197	bannister	3	0.026	hovel	0	0
box	124	1.076	towel	2	0.017	hill	0	0
fan	91	0.79	sink	2	0.017	grandstand	0	0
clock	79	0.685	pool table	2	0.017	fountain	0	0
blanket	79	0.685	mountain	2	0.017	food	0	0
chandelier	78	0.677	house	2	0.017	flag	0	0
plate	77	0.668	grass	2	0.017	field	0	0
sconce	74	0.642	fence	2	0.017	escalator	0	0
blind	66	0.573	case	2	0.017	earth	0	0
tray	63	0.547	building	2	0.017	dirt track	0	0
railing	58	0.503	washer	1	0.009	countertop	0	0
basket	57	0.495	toilet	1	0.009	conveyer belt	0	0
stool	54	0.469	sea	1	0.009	car	0	0
bookcase	47	0.408	palm	1	0.009	canopy	0	0
bottle	43	0.373	dishwasher	1	0.009	bus	0	0
column	38	0.33	cradle	1	0.009	bulletin board	0	0
sculpture	36	0.312	bicycle	1	0.009	bridge	0	0
step	29	0.252	base	1	0.009	booth	0	0
glass	29	0.252	ashcan	1	0.009	bathtub	0	0
desk	24	0.208	arcade machine	1	0.009	barrel	0	0
stairway	22	0.191	waterfall	0	0	awning	0	0
tree	17	0.148	water	0	0	apparel	0	0
wardrobe	15	0.13	van	0	0	animal	0	0
pillow	15	0.13	truck	0	0	airplane	0	0

SUPPLEMENTARY TABLE 6 – BEDROOM MODEL

Class Name	Count	Percentage	Class Name	Count	Percentage	Class Name	Count	Percentage
wall	1388	8.913	swivel chair	21	0.135	waterfall	0	0
bed	1353	8.688	plate	20	0.128	water	0	0
floor	1275	8.187	bag	17	0.109	washer	0	0
table	1038	6.665	poster	16	0.103	van	0	0
lamp	1004	6.447	bench	16	0.103	truck	0	0
ceiling	809	5.195	sky	15	0.096	traffic light	0	0
painting	802	5.15	tree	14	0.09	trade name	0	0
windowpane	794	5.099	sculpture	13	0.083	tower	0	0
pillow	698	4.482	step	12	0.077	tent	0	0
curtain	651	4.18	apparel	12	0.077	tank	0	0
cushion	556	3.57	seat	11	0.071	streetlight	0	0
door	355	2.28	person	11	0.071	stage	0	0
chair	280	1.798	column	11	0.071	skyscraper	0	0
cabinet	277	1.779	bathtub	11	0.071	sidewalk	0	0
chest of drawers	263	1.689	ashcan	11	0.071	ship	0	0
mirror	252	1.618	sink	9	0.058	screen	0	0
rug	204	1.31	cradle	8	0.051	sand	0	0
book	194	1.246	toilet	7	0.045	runway	0	0
vase	190	1.22	stairs	7	0.045	road	0	0
wardrobe	187	1.201	computer	7	0.045	river	0	0
clock	186	1.194	countertop	6	0.039	pool table	0	0
armchair	185	1.188	sea	5	0.032	pier	0	0
plant	184	1.182	screen door	4	0.026	path	0	0
sconce	173	1.111	monitor	4	0.026	oven	0	0
flower	173	1.111	ball	4	0.026	minibike	0	0
light	165	1.06	swimming pool	3	0.019	lake	0	0
box	143	0.918	stairway	3	0.019	kitchen island	0	0
fan	128	0.822	grass	3	0.019	hovel	0	0
television receiver	119	0.764	earth	3	0.019	hill	0	0
shelf	119	0.764	building	3	0.019	grandstand	0	0
pot	101	0.649	signboard	2	0.013	fountain	0	0
blind	85	0.546	shower	2	0.013	food	0	0
ottoman	81	0.52	rock	2	0.013	field	0	0
desk	79	0.507	refrigerator	2	0.013	escalator	0	0
sofa	78	0.501	mountain	2	0.013	dishwasher	0	0
basket	77	0.494	microwave	2	0.013	dirt track	0	0
coffee table	63	0.405	flag	2	0.013	crt screen	0	0
plaything	59	0.379	boat	2	0.013	counter	0	0
blanket	57	0.366	animal	2	0.013	conveyer belt	0	0
radiator	56	0.36	stove	1	0.006	case	0	0
tray	50	0.321	pole	1	0.006	car	0	0
bottle	49	0.315	palm	1	0.006	bus	0	0
stool	47	0.302	land	1	0.006	bulletin board	0	0
fireplace	44	0.283	house	1	0.006	buffet	0	0
chandelier	42	0.27	hood	1	0.006	bridge	0	0
glass	36	0.231	fence	1	0.006	booth	0	0
towel	35	0.225	bicycle	1	0.006	barrel	0	0
canopy	27	0.173	base	1	0.006	bar	0	0
railing	24	0.154	bannister	1	0.006	arcade machine	0	0
bookcase	22	0.141	awning	1	0.006	airplane	0	0
